# Risk of postpartum hemorrhage with increasing first stage labor duration

**DOI:** 10.1038/s41598-024-72963-2

**Published:** 2024-09-27

**Authors:** Linnea V. Ladfors, Xingrong Liu, Anna Sandström, Louise Lundborg, Alexander J. Butwick, Giulia M. Muraca, Jonathan M. Snowden, Mia Ahlberg, Olof Stephansson

**Affiliations:** 1 Clinical Epidemiology Division, Department of Medicine, Karolinska Institutet, Solna, Stockholm, Sweden; 2 Department of Women’s Health, Division of Obstetrics, Karolinska University Hospital, Stockholm, Sweden; 3 Dept. of Anesthesiology, Perioperative, and Pain Medicine, Stanford University School of Medicine, Stanford, CA USA; 4 Departments of Obstetrics and Gynecology and Health Research Methods, Evidence & Impact, Faculty of Health Sciences, McMaster University, Hamilton, ON Canada; 5 School of Public Health, Oregon Health & Science University - Portland State University, Portland, OR USA

**Keywords:** Epidemiology, Risk factors

## Abstract

With increasing rates of postpartum hemorrhage (PPH) in high-income countries, an important clinical concern is the impact of labor duration on the risk of PPH. This study examined the relationship between increasing active first stage labor duration and PPH and explored the role of second stage labor duration and cesarean delivery (CD) in this association. Including 77,690 nulliparous women with spontaneous labor onset, first stage labor duration was defined as the time from 5 cm to 10 cm, second stage duration from 10 cm dilation to birth and PPH as estimated blood loss > 1000 ml. Using modified Poisson regression for risk ratios (RR) and confidence intervals (CI), we found a 1.5-fold (RR, 1.53; 95% CI, 1.41‒1.66) increased PPH risk when first stage of labor exceeded 12.1 h compared to the reference (< 7.7 h). Mediation analysis showed that 18.5% (95% CI, 9.7‒29.6) of the increased PPH risk with a prolonged first stage (≥ 7.7 h) was due to a prolonged second stage (> 3 h) or CD. These results suggest that including first stage duration in intrapartum assessments could improve PPH risk identification in first-time mothers with a singleton fetus in vertex presentation at full term with spontaneous labor onset.

**Supplementary Information:**

The online version contains supplementary material available at 10.1038/s41598-024-72963-2.

## Introduction

 Postpartum hemorrhage (PPH), a major cause of severe maternal morbidity, is occurring with increasing frequency in high-income countries^1^ and an important clinical concern is the impact of prolonged labor duration on the risk of PPH. Although a prolonged second stage of labor has been identified as a risk factor for PPH^2^ and is included in national guidelines and risk assessment tools^3,4^ weaker evidence exist detailing the relationship between first stage labor duration and PPH risk^5–7^.

There is emerging evidence for a strong association between first stage labor duration and second stage duration and mode of delivery^8,9^. However, the magnitude and the proportion of the association between first stage duration and PPH that could be attributed to a prolonged second stage and/or an increased risk of cesarean delivery (CD) is unknown. To optimize current clinical approaches for PPH risk assessment, examining the direct effect of a prolonged first stage on PPH risk could be valuable^4^.

Recent population-based data indicate that almost one third of PPH cases in Sweden occur in nulliparous, singleton, term, vertex pregnancies with spontaneous labor onset, demonstrating that risk factors for PPH in this group of women is important to examine further^10^.

Therefore, the study objective was to investigate the relationship between increasing active first stage duration and PPH, in nulliparous women with spontaneous labor onset, and to explore the potential joint mediated effect of a prolonged second stage and/or CD in this association in a large population-based cohort.

## Methods

A population-based study using the Stockholm-Gotland Perinatal Cohort^11^, established in 2008, which contains prospectively collected data from antenatal and delivery care on all singleton births in Stockholm and Gotland. Information, including data on labor progression and timepoints for interventions from the partograph, is automatically transferred from the electronic medical records (Obstetrix, Cerner Inc)^11^. In Sweden, antenatal and delivery care is free of charge, the primary caregivers during pregnancy and childbirth are midwives and > 99% of deliveries are hospital-based^12^. Cervical examinations during labor are routinely performed by midwives and entered into the partograph. The scope of the study was the progressing period of the active first stage of labor and to harmonize with the World Health Organization definition and prior publications^13,14^ the onset of active first stage of labor was defined as the timepoint of 5 cm cervical dilation.

**Fig. 1:**
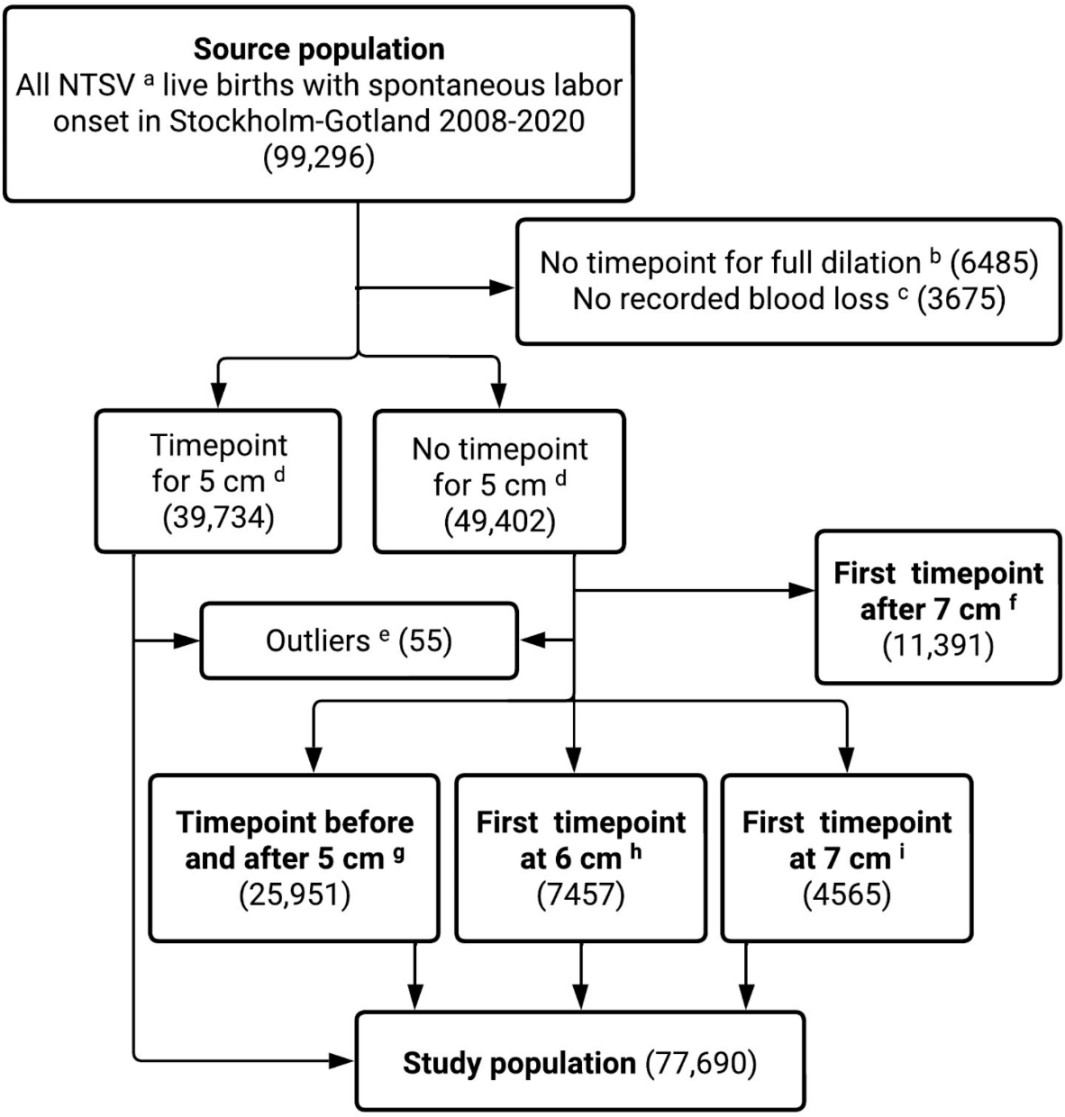
Flowchart for selection of the study population. Nulliparous term (≥ 37 weeks gestation) singleton vertex deliveries. Endpoint for first stage of labour. *N* = 4573 were cesarean deliveries. Missing data on estimated blood loss after delivery. For measuring the exposure, first stage labor duration, the onset of first stage was defined as the first recorded timepoint of 5 cm cervical dilation. First stage shorter than 0 min or first stage longer than 25 h. First timepoint at 8 cm: *n* = 3176; at 9 cm: *n* = 1711; at 10 cm: *n* = 6504. The 5 cm timepoint was not imputed for these women. Imputed timepoint for 5 cm cervical dilation using available timepoints for 3–4 cm and from 6-full cervical dilation. Imputed timepoint for 5 cm cervical dilation using available timepoints for 6 cm and full cervical dilation. Imputed timepoint for 5 cm cervical dilation using available timepoints for 7 cm and full cervical dilation.

Our source population was nulliparous women with live, singleton, term (≥ 37 weeks gestation) births, in vertex presentation with spontaneous labor onset between January 1, 2008 and June 15, 2020, *n* = 99,296. We excluded women with missing data on the endpoint of first stage (full cervical dilation), women with a first stage intrapartum CD, and with missing data on estimated blood loss (Fig. 1, *n* = 10,160). The timepoint for 5 cm dilation was recorded for 39,734 women. For the 49,402 women with missing notations for 5 cm but with recorded timepoints before and after 5 cm or at 6–7 cm, imputation of the timepoint for 5 cm was perfomed and these women were included in the study. A weighting approach was used for the imputation, based on the cumulative duration data from the study by Lundborg et al.^15^ As an example, for women with recorded timepoints at 4 and 6 cm, their timepoint for 5 cm was estimated from: the recorded timepoint of 4 cm + the estimated weight * the individual time difference between 4 and 6 cm. This imputation strategy has been used in prior work^14^ and is further descibed in Supplementary Methods and Supplementary Figure S1-2. The timepoint for 5 cm was not imputed for women with a first notation of cervical dilation after 7 cm (*n* = 11,391) and these women were not included in the study population. After exclusion of women with implausible first stage durations (< 0 min or > 25 h, *n* = 55) the final study population consisted of 77,690 deliveries (Fig. 1).

The continuous exposure, first stage duration, was defined as the time interval between 5 cm cervical dilation and the endpoint for first stage, i.e. the first recorded timepoint for full cervical dilation. First stage duration was categorized into percentiles (< 75th, 75th ‒ <90th, 90th ‒ <95th and ≥ 95th ). Second stage duration was defined as the time interval between the first notation of full cervical dilation and birth and classified into percentiles (< 75th, 75th ‒ <90th, 90th ‒ <95th and ≥ 95th ).

Based on previous findings, indicating a relationship between first stage duration and second stage duration and CD^8^ as well as an association between increasing duration of the second stage and both CD and PPH^16^ we posited an underlying causal structure with second stage duration and CD as intermediates on the pathway from first stage duration to PPH (Supplementary Figure S3).

The outcome was PPH, defined as estimated blood loss > 1000 ml until 2 h after delivery, the Swedish definition during the study period^17^. Procedures for quantification and reporting of blood loss in Swedish delivery wards is described elsewhere^18^.

Maternal age at delivery, early pregnancy body mass index (BMI; kg/m^2^), infant birthweight, oxytocin augmentation before 10 cm dilation (yes vs. no), year of delivery and delivery hospital (*n* = 8) were identified as confounders for the association between first stage duration and PPH using a directed acyclic graph (Figure S3). The continuous variables, maternal age, BMI and infant birth weight, were mean centered and included in multivariable analyses with a quadratic term to account for non-linearity. In all multivariable analyses complete case analysis was applied.

The distribution of maternal, labor and infant characteristics was described by first stage duration percentile category. Temporal trend in the rate of PPH was recorded over the study period. Logistic regression was used to estimate the crude proportion of deliveries with PPH by increasing first stage duration (h), which was modelled as a natural cubic spline function^19^. Based on a scatter plot of first stage duration and the predicted adjusted risk of PPH for each delivery, a median risk curve with 95% confidence intervals (CI) of risk was estimated from quantile regression and compared to the estimated crude proportion of PPH deliveries. Next, we estimated crude and adjusted risk ratios (RRs) with 95% CIs between first stage duration percentile (with < 75th percentile as reference) and PPH using modified Poisson regression with robust standard errors. Since oxytocin augmentation before 10 cm dilation could be regarded as both a confounder and a mediator, influenced by the labor progress during the first stage of labor, we ran separate models excluding and including this predictor in the multivariable analysis.

To examine the proportion of the association between a prolonged first stage of labor (≥ 75th percentile) and PPH that was due to a prolonged second stage (≥ 75th percentile) or CD, mediation analysis was performed modelling a prolonged second stage and CD as joint mediators (Supplementary Methods)^20^. We estimated the natural direct effect of a prolonged first stage on PPH and the jointly mediated effect through a prolonged second stage and CD^21^. The mediated effect is interpretable as the effect of a prolonged first stage on PPH that can be attributed to a prolonged second stage or CD; and the natural direct effect corresponds to the effect of a prolonged first stage on PPH “directly” (i.e. through all other pathways). The proportion mediated, i.e. the proportion of the total effect of a prolonged first stage on PPH that is mediated by a prolonged second stage/CD was estimated on the risk difference scale^21,22^.

To assess the robustness of our findings we performed three sensitivity analyses. First, to examine potential bias introduced with our imputation approach, we calculated RRs in the population with a recorded 5 cm timepoint, i.e. excluding those with an imputed timepoint for 5 cm. Second, to investigate if the percentile categorization could influence the estimates, the Poisson regression was performed using different categorizations and reference groups (< 50th and 60th percentile) of first stage duration (Supplementary Table S1-S2). Last, to assess the risk of selection bias, characteristics and rates of PPH were compare d between the study population and the 11,391 excluded deliveries in which the first recorded timepoint for cervical dilation was greater than 7 cm.

SAS version 9.4 (SAS Institute, Cary, NC) and R Statistical Software (v4.1.3; 2022-03-10) were used for data management and analysis^23^. The study did not include any patient or public involvement and no core outcome set was used. Permission for the study was obtained from the Swedish Ethical Review Authority (2009/275 − 31, 2012/365 − 32, 2019–02818). The research was performed according to regulations and all data was pseudonymized prior to analysis.

## Results

**Fig. 2:**
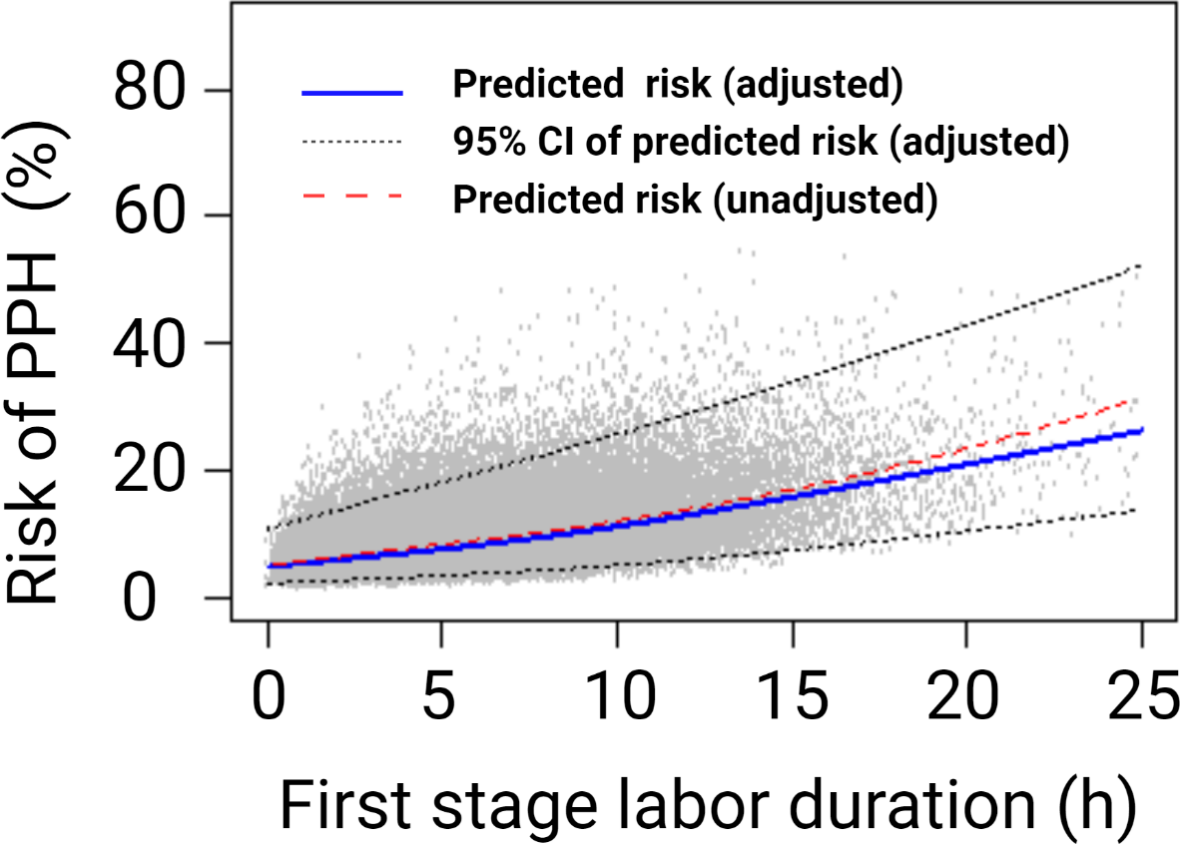
Scatter plot of the association between first stage labor duration (h) and unadjusted (dashed red line) and adjusted (blue line) risk of postpartum hemorrhage (PPH; >1000 ml) in the study population,*n* = 77,690. Confidence intervals (CI, dotted black line). The model was adjusted for maternal age, early pregnancy body mass index, delivery clinic, year of delivery, oxytocin < 10 cm and birth weight.

Our study population comprised 77,690 deliveries, of which *n* = 6880 (8.8%; 95% CI, 8.6‒9.0) had PPH. The estimated median regression curve between the first stage duration (h), modelled using restricted cubic splines, and the predicted risk of PPH with 95% CI indicate a linear relationship between first stage duration and increasing PPH risk (Fig. 2).

Maternal, obstetric and infant characteristics in the study population, categorized by first stage duration percentiles, are presented in Table 1. increased rates of PPH from 7.9 to 10.7% over the study period (Supplementary Table S3). Rates of missingness were very low (< 2%) for all variables except BMI, for which 4% of deliveries had incomplete information. Compared with women with shorter first stage duration, women with longer first stage duration more often had oxytocin augmentation of labor, operative delivery and infants with a birth weight ≥ 4000 g.

**Table 1:** Maternal and labor characteristics of singleton deliveries in the study population (*n* = 77,690), nulliparous women at term with the infant in vertex position and spontaneous onset of labor, stratified by percentile categories of first stage labor duration.

	First stage labor duration			
Percentile of first stage labor duration First stage (h)	< 75th < 7.7	75th ‒ <90th 7.7 ‒ <10.3	90th ‒ <95th 10.3 ‒ <12.1	≥ 95th ≥ 12.1
All deliveries	58,235 (75.0)	11,670 (15.0)	3896 (5.0)	3889 (5.0)
Maternal age (years)	29 (26‒32)	30 (27‒33)	30 (27‒33)	30 (28‒33)
< 30	30,698 (52.7)	5518 (47.3)	1719 (44.1)	1613 (41.5)
30‒34	20,277 (34.8)	4475 (38.4)	1568 (40.2)	1618 (41.6)
35+	7232 (12.4)	1676 (14.4)	607 (15.6)	658 (16.9)
Missing	28	1	2	.
Early pregnancy BMI (kg/m2)	22.6 (20.8‒25.0)	22.7 (20.9‒25.3)	22.9 (21.0‒25.2)	23.0 (21.0‒25.4)
< 25.0	42,103 (72.3)	8208 (70.3)	2749 (70.6)	2692 (69.2)
25.0‒29.9	10,330 (17.7)	2239 (19.2)	758 (19.5)	781 (20.1)
30.0 +	3467 (6.0)	787 (6.7)	255 (6.6)	276 (7.1)
Missing	2335 (4.0)	436 (3.7)	134 (3.4)	140 (3.6)
Smoking 1st antenatal visit	2217 (3.8)	325 (2.8)	104 (2.7)	89 (2.3)
Missing	804 (1.4)	160 (1.4)	66 (1.7)	61 (1.6)
Cohabitation	52,488 (90.1)	10,665 (91.4)	3563 (91.4)	3560 (91.5)
Missing	1032 (1.8)	213 (1.8)	71 (1.8)	71 (1.8)
IVF/ICSI treatment	3116 (5.4)	685 (5.9)	243 (6.2)	257 (6.6)
Epidural analgesia	36,815 (63.2)	9619 (82.4)	3342 (85.8)	3375 (86.8)
Augmentation of labor with oxytocin started				
No oxytocin	22,513 (38.7)	1298 (11.2)	237 (6.1)	159 (4.1)
< 5 cm	5380 (9.2)	629 (5.4)	209 (5.4)	159 (4.1)
5–10 cm	13,951 (24.0)	7518 (64.4)	3005 (77.1)	3232 (83.1)
> 10 cm	16,391 (28.2)	2225 (19.1)	445 (11.4)	339 (8.7)
Second stage duration (h)				
< 3.0	45,660 (78.4)	7690 (65.9)	2468 (63.4)	2394 (61.6)
3.0‒<4.1	7706 (13.2)	2322 (19.9)	816 (20.9)	790 (20.3)
4.1‒<4.7	2509 (4.3)	826 (7.1)	277 (7.1)	336 (8.6)
≥ 4.7	2360 (4.0)	832 (7.1)	335 (8.6)	369 (9.5)
Mode of delivery				
Spontaneous vaginal delivery	49,697 (85.3)	8849 (75.8)	2696 (69.2)	2470 (63.5)
Operative vaginal delivery	7468 (12.8)	2255 (19.3)	905 (23.2)	987 (25.4)
CD (second stage)	1070 (1.8)	566 (4.8)	295 (7.6)	432 (11.1)
Post‒term gestation (> 42 weeks)	2164 (3.7)	644 (5.5)	242 (6.2)	261 (6.7)
Infant birth weight ≥ 4000 g	5805 (10.0)	1921 (16.5)	785 (20.2)	903 (23.2)

Results presented as median (interquartile range) and n (column percent). BMI, body mass index; IVF, in vitro fertilization; ICSI, intracytoplasmic sperm injection; CD, cesarean delivery.

Compared to labors with a first stage duration < 7.7 h (75th percentile), labors with a first stage duration between 10.3 and 12.1 h (90th to < 95th percentile) and ≥ 12.1 h (95th percentile) had a 37% (adjusted RR 1.37, 95% CI, 1.25–1.49) and 53% increased risk of PPH (adjusted RR 1.53, 95% CI, 1.41‒1.66), respectively (Table 2). Excluding oxytocin before 10 cm dilation from the adjusted model resulted in a slight increment in PPH estimates.

**Table 2:** Absolute risks of postpartum hemorrhage (PPH) and unadjusted and adjusted risk ratios (RR) for PPH with increasing percentile categories of first stage labor duration in the study population, *n* = 77,690.

	First stage labor duration			
Percentile of first stage labor duration First stage (h)	< 75th < 7.7	75th ‒ <90th 7.7 ‒ <10.3	90th ‒ <95th 10.3 ‒ <12.1	≥ 95th ≥ 12.1
Absolute risk, n (%)	4411 (7.6)	1274 (10.9)	541 (13.9)	654 (16.8)
Unadjusted RR (95% CI)	1.00 (ref)	1.44 (1.36‒1.53)	1.83 (1.69‒1.99)	2.22 (2.06‒2.39)
Adjusted RR* (95% CI)	1.00 (ref)	1.17 (1.10‒1.24)	1.37 (1.25‒1.49)	1.53 (1.41‒1.66)
Adjusted RR** (95% CI)	1.00 (ref)	1.24 (1.16‒1,31)	1.48 (1.36‒1.61)	1.66 (1.53‒1.79)

*Maternal age, maternal pre-pregnancy BMI, oxytocin started before 10 cm cervical dilation, birth weight, year of delivery and delivery clinic.

** Excluding oxytocin started before 10 cm dilation.

*n* = 3102/77,690 deliveries were excluded from the adjusted analysis due to missing data on confounder variables.

In Supplementary Fig. S4, absolute risks of PPH per first stage duration percentile (panel A-D) and second stage duration percentile (X-axes) are presented. The highest absolute risk of PPH (25.2%) was observed in women with a first (≥ 12.1 h) and second (≥ 4.7 h) stage labor duration above the 95th percentiles.

In mediation analysis, modelling a prolonged second stage of labor (≥ 3.0 h) and CD as joint mediators, the direct effect of a prolonged first stage duration (≥ 7.6 h) on the risk of PPH was 1.25 (95% CI, 1.15‒1.36) while the proportion of the association between a prolonged first stage and PPH that was mediated jointly through a prolonged second stage and CD was 18.5% (95% CI, 9.7‒29.6; Supplementary Table S4).

When rerunning the main analysis in the population with a recorded timepoint for 5 cm, the estimates were consistent with those from the study population (Supplementary Table S5). The same trend with increasing PPH risk was apparent in higher percentile categories compared to the designated reference category when varying the categorization of the exposure (Supplementary Table S1-S2). Women with a first timepoint for dilation after 7 cm (*n* = 11,391, Fig. 1) had lower rates of oxytocin augmentation, operative delivery and infant birth weights ≥ 4000 g compared to the study population while rates of PPH were similar in both groups (Supplementary Table S6).

## Discussion

In this cohort of 77,690 nulliparous women with term, singleton pregnancies and spontaneous labor onset who underwent vaginal delivery or CD in the second stage of labor, we observed a positive association between the first stage duration and PPH risk. Compared to deliveries with a first stage duration < 75th percentile (7.7 h), deliveries with a first stage duration ≥ 75th percentile (12.1 h) had a 53% higher risk of PPH. Mediation analysis revealed that approximately 18% of the increase in risk of PPH with a prolonged first stage was due to a prolonged second stage or CD in the second stage of labor. Based on our study findings, a prolonged first stage of labor is an important PPH risk factor and incorporating this factor into intrapartum risk assessment could improve the identification of nulliparous women with spontaneous onset at high risk for PPH.

While a prolonged second stage of labor has been associated with an increased risk of PPH^2,24^ prior studies examining the association between the first stage labor duration and PPH risk have reported null results or weak estimates^5–7^. Cheng et al.^5^ analyzed nulliparous births in California and did not observe an association between first stage duration > 30 h and PPH after adjustment for confounders including second stage duration and mode of delivery (adjusted odds ratio: 1.18, 95% CI, 0.95–1.45). In a single center study, Blankenship et al.^6^ examined associations between first stage duration (4 to 10 cm dilation) and PPH and reported a small increase in risk (odds ratio: 1.8, 95% CI:1.1–2.9) in women with a first stage duration ≥ 90th compared with < 90th percentile. However, the study did not report which confounders that had been included in multivariable analysis and estimates were not presented separately for induction of labor vs. spontaneous labor onset. This discrepancy as compared to our findings may be due to variations in the definition of onset of first stage and the use of different cut-offs for ‘prolonged labor’ but it may also be explained by previous studies treating second stage duration and mode of delivery as confounders instead of intermediates in the association between first stage duration and PPH. This study is, to our knowledge, the first to date to investigate effects of first stage duration and second stage duration as two separate but linked entities. The findings from the mediation analysis indicate that risks previously ascribed to a prolonged second stage of labor might, to a significant extent, originate from a longer first stage. Previous positive findings for the relationship between second stage and PPH as compared to null findings for associations between first stage duration and PPH, could be explained by difficulties in defining the onset of the first stage of labor, over adjustment for intermediates or insufficient data on the first stage of labor and timing of associated interventions^5–7^.

Factors explaining the association between increasing first stage duration and PPH could be biological or related to labor management. A longer first stage duration, with many hours of uterine contractions, may influence the adequacy of myometrial contractility after delivery thereby increasing the risk of atonic hemorrhage. Similarly, slower labor progress might necessitate oxytocin augmentation, which, in high doses, is presumed to desensitize myometrial oxytocin receptors^25^. There is conflicting evidence on the relationship between labor duration, oxytocin augmentation and PPH^26,27^. One recent study, based on data from a randomized controlled trial, reported an independent association between high doses of oxytocin (≥ 20 mU/minute) and PPH^26^. Conversely, no association between oxytocin use and PPH was found in a newly published study using a rigorous propensity score-based approach to control for confounders, including the pattern of labor progression before the initiation of oxytocin augmentation^27^. In our study, the highest absolute risk of PPH was found in women with a first stage duration ≥ 12.1 h and among these, 92% received oxytocin augmentation. It is important to recognize that while a longer duration of the first stage of labor can be factored into the risk assessment for PPH, it should not be construed as a recommendation to perform a cesarean delivery earlier during labor. Such an approach could inadvertently increase the overall cesarean delivery rate, which itself is associated with a higher risk of PPH^28^.

The main strengths of our study include the population-based, real-world data on labor duration including longitudinal timepoints for cervical dilation and initiation of oxytocin augmentation. Another important strength is our mediation analysis, evaluating the potential role of a prolonged second stage and CD on the pathway between first stage duration and PPH risk. Most previous studies investigating the relationship between first stage duration and PPH have controlled for mode of delivery or second stage labor duration using regression-based adjustment or by restricting the analysis to vaginal deliveries^5,7^. This approach has limitations since, chronologically, the second stage occurs after the first stage of labor and the decision to perform a CD can be a consequence of a long first or second stage of labor. Lastly, we performed several sensitivity analyses to assess the robustness of our findings in which the association between increasing first stage duration and PPH was consistent, strengthening the reliability of our results,

Our study has several limitations. The joint mediation approach does not allow for more than two mediators, and therefore we could not account for any mediated effect through CD vs. operative vaginal or spontaneous vaginal delivery. We did not examine the relationship between first stage duration and PPH by etiology or differentiate between PPH in CD or in vaginal delivery. The decision to not stratify our analysis on PPH etiology was taken a priori, since surgical bleeding could only occur after a CD and conditioning on the etiology for PPH would be exchangeable to stratifying on the intermediate mode of delivery.

Our results cannot be generalized to women who undergo CD before 10 cm cervical dilation (first stage CD) because these women were excluded from our study. This approach has been used in other studies examining first stage duration and adverse outcomes^6,29^. The rate of CD among deliveries with missing information on full cervical dilatation was low in our population of nulliparous, term, vertex, singleton pregnancies with spontaneous labor onset (4573/99,296; 4.6%). This could limit the generalizability of our findings to other high-income countries with higher rates. Likewise, our findings cannot be generalized to parous women or women undergoing induction of labor. Lastly, it is possible that estimation of blood loss has become more thorough and that labor guidelines have been updated over the study period. We found PPH increased over this time. To account for these potential variations, we adjusted for year of delivery and delivery hospital in our multivariable analysis, however, the potential for residual confounding cannot be dismissed.

Compared to women with longer labors, women with rapid first stage labor progress may have fewer cervical examinations and be more likely to have missing data in the partograph on the timepoint for 5 cm cervical dilation, i.e. missing not at random. We addressed this potential source of bias by imputing the timepoint for 5 cm in women missing this timepoint which may have introduced bias for exposure assessment. Reassuringly, there were no important differences in PPH risk estimates with increasing first stage duration in women with a recorded 5 cm examination as compared to the study population. Our findings are also limited to women admitted to the delivery ward before 8 cm dilation. Importantly, the rate of PPH among women admitted after 7 cm dilation was comparable to those of the study population. Considering the high rates of oxytocin augmentation in women with longer first stage labor duration and the observational design of this study our findings should not be interpreted as an indication for labor augmentation when the first stage duration exceeds a time-based ‘cut-point’ to prevent PPH. Lastly, since this is an observational study, it cannot be ruled out that there are unmeasured confounders, influencing both first stage duration and PPH.

In conclusion, increasing first stage of labor duration is an independent risk factor for PPH in nulliparous women with term, singleton pregnancy with vertex presentation and spontaneous labor onset, which should be considered in risk assessment for PPH.

## Electronic supplementary material

Below is the link to the electronic supplementary material.

Supplementary Material 1

## Data Availability

Data AvailabilityThe Stockholm Gotland Perinatal Cohort was used for this study. Information in the database was retrieved from the medical record system Obstetrix. The database is stored in the Clinical Epidemiology Division at Karolinska Institutet Stockholm, Sweden. Public data sharing from this database is not permitted. However, any researcher can access the data by obtaining an ethical approval from a regional ethical review board and are available from the corresponding author on reasonable request.
