# Supplementary material for: Risk of postpartum hemorrhage with increasing first stage labor duration
Source: Sci Rep. 2024 Sep 27;14:22152. doi: 10.1038/s41598-024-72963-2 (PMC11436723; doi:10.1038/s41598-024-72963-2)
Supplement: Supplementary file 1 — Supplementary Material 1 [file 41598_2024_72963_MOESM1_ESM.pdf]

Supplementary Methods.

Imputation strategy

Since the notation of 5 cm dilation, i.e. onset of first stage of labor, was missing in the partograph for a substantial proportion of the source population, the timepoint for a cervical dilation of 5 cm was estimated and imputed accordingly to minimize the risk of selection bias and improve precision. Imputation for the 5 cm timepoint was performed 1) for women with measurements for 3 or 4 cm cervical dilation and at least one measurement between 6 and 10 cm (pattern 1-10, Supplementary Figure S1) and 2) for women with a first recorded timepoint at 6 or 7 cm. The cumulative duration data from the study by Lundborg et al.[1] was used for weighting. Through the internal validation by those with known notation of 5 cm, we could observe approximated Gaussian distribution of time difference between observed and imputed predicted timepoints for 5 cm by the proposed weights for pattern 1-3 and 6-11 while pattern 4-5 and 12 were slightly right-skewed, Supplementary Figure S2.

Imputation method with example:

Imputation of timepoint for 5 cm with one notation recorded before (4 cm) and one after (6 cm) 5 cm. (e.g. pattern 1; Supplemental Figure 1).

$$\begin{aligned} &Imputed\ datetime\ of\ 5\ cm \\ &\quad = known\ datetime\ of\ 4\ cm + estimated\ \boldsymbol{weight} \\ &\quad * individual\ time\ difference\ between\ 4\ and\ 6\ cm \end{aligned}$$

Here, each weight is separately derived from the population-based cumulative durations of labor from one cervical dilation to 10 cm. For example, 56.5%= (5.33-3.9)/ (5.33-2.8) is calculated for nulliparous women in pattern 1 (Supplemental Figure S1), where the median cumulative durations were estimated to be 5.33 hours, 3.90 hours, and 2.80 hours from cervical dilation of 4 cm, 5 cm, and 6 cm to 10 cm, respectively [1].

Rationale for joint mediation approach

From Supplemental Figure 3 it can be deduced that second stage duration (L) is an exposure-induced mediator-outcome confounder of the relationship between first stage of labor duration, cesarean delivery and postpartum hemorrhage. This violates one of the assumptions for traditional mediation analysis and performing regular mediation analysis would result in biased estimates [2]. To enable the calculation of natural direct effects (NDE) and natural indirect effects (NIE) in this case a joint mediator approach was deemed the most appropriate. With this approach, M (cesarean delivery) and L (prolonged second stage duration; Supplemental Figure 3) can be considered as joint mediators [3]. First, weighting-

based estimators, derived from inverse probability weighting based on logistic regression models for A, M and L were obtained. Subsequently, the NDE of a prolonged first stage ($\geq75^{th}$ percentile) on PPH and the NIE mediated through: a) a prolonged second stage ($\geq75^{th}$ percentile), or b) CD or c) a prolonged second stage through CD, were estimated. For assumptions to hold this analysis required controlling for confounders of the relationship between first stage and CD, between first stage and second stage as well as for the confounders of CD and PPH and second stage and PPH. As in the main analysis, these confounders included maternal age, early pregnancy BMI and infant birth weight, oxytocin before 10 cm dilation (yes vs no), delivery clinic and year of delivery (Supplemental Figure 3). The proportion mediated was calculated as OR_{NDE} *(OR_{NIE}-1) /OR_{NDE}*OR_{NIE}-1, [4] with the ORs obtained from the joint mediation analysis [5]. The bootstrap method with replacement was used to construct confidence intervals for the NDE, NIE and the proportion mediated. This meant repeating the mediation analysis including the inverse probability weighting for 1000 samples [3].

References

1 Lundborg, L. *et al.* First stage progression in women with spontaneous onset of labor: A large population-based cohort study. *PloS one* **15**, e0239724, doi:10.1371/journal.pone.0239724 (2020).

2 Avin, C., Shpitser, I. & Pearl, J. *Identifiability of Path-Specific Effects*. Vol. 19 (2005).

3 Vanderweele, T. J., Vansteelandt, S. & Robins, J. M. Effect decomposition in the presence of an exposure-induced mediator-outcome confounder. *Epidemiology (Cambridge, Mass.)* **25**, 300-306, doi:10.1097/ede.0000000000000034 (2014).

4 Ananth, C. V. & Brandt, J. S. A principled approach to mediation analysis in perinatal epidemiology. *American journal of obstetrics and gynecology* **226**, 24-32.e26, doi:10.1016/j.ajog.2021.10.028 (2022).

5 Vanderweele, T. J. & Vansteelandt, S. Odds ratios for mediation analysis for a dichotomous outcome. *American journal of epidemiology* **172**, 1339-1348, doi:10.1093/aje/kwq332 (2010).

Supplementary Figure S1. Imputation patterns for onset of first stage of labor, timepoint for 5 cm cervical dilation), weights and root mean square errors (RMSE).

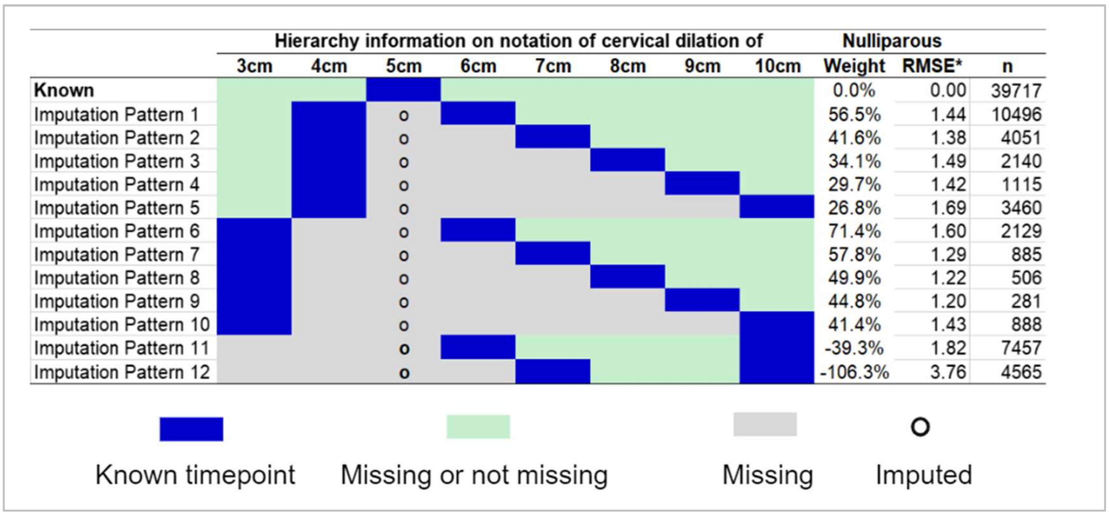

Supplementary Figure S2. Distribution of differences (h) between observed timepoints for cervical dilation and predicted timepoints for cervical dilation by imputation pattern (1-12). Blue lines indicating the normal distribution.

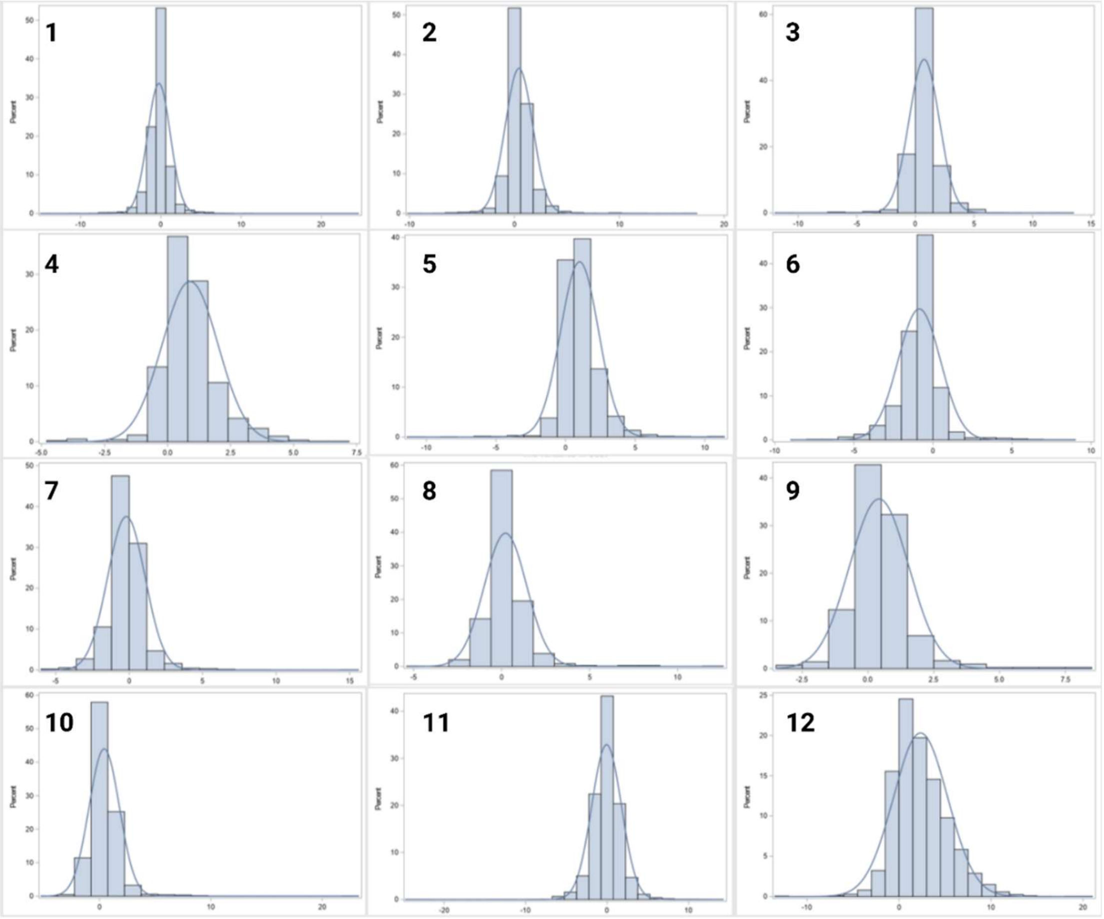

Supplementary Figure S3. Directed acyclic graph illustrating the suggested relationship between our exposure: first stage duration (A), the mediators: second stage duration (L) and cesarean delivery (CD, M), the confounders and our outcome postpartum hemorrhage (PPH, Y) depicting potential pathways for direct (DE) and indirect effects (IE) of a prolonged first stage of labor on PPH. Maternal age, body mass index (BMI), delivery clinic, year of delivery, oxytocin <10 cm dilation and fetal birthweight are all considered common causes of both exposure and outcome since these may impact labor progress and labor management and can influence the risk of PPH. Mediators are variables positioned on the causal pathway between first stage duration and PPH and include second stage duration and CD. Oxytocin <10 cm dilation could also be considered a mediator since progress during first stage of labor may influence the use of oxytocin for labor augmentation.

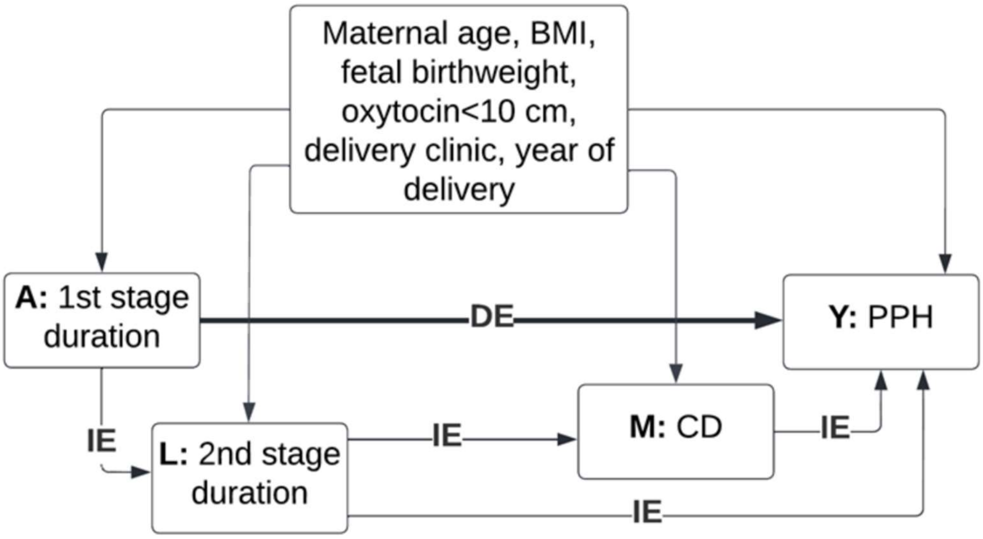

Supplementary Figure S4. Proportion with postpartum hemorrhage (PPH) with error bars indicating the 95% confidence intervals, with increasing second stage of labor duration in the study population (n=77,690) stratified by percentiles of first stage labor duration, panel A) first stage<7.7 h; B) 7.7–<10.3 h; C) 10.3–<12.1 h; D) ≥12.1 h. n with PPH/N indicated in bold above the corresponding bar.

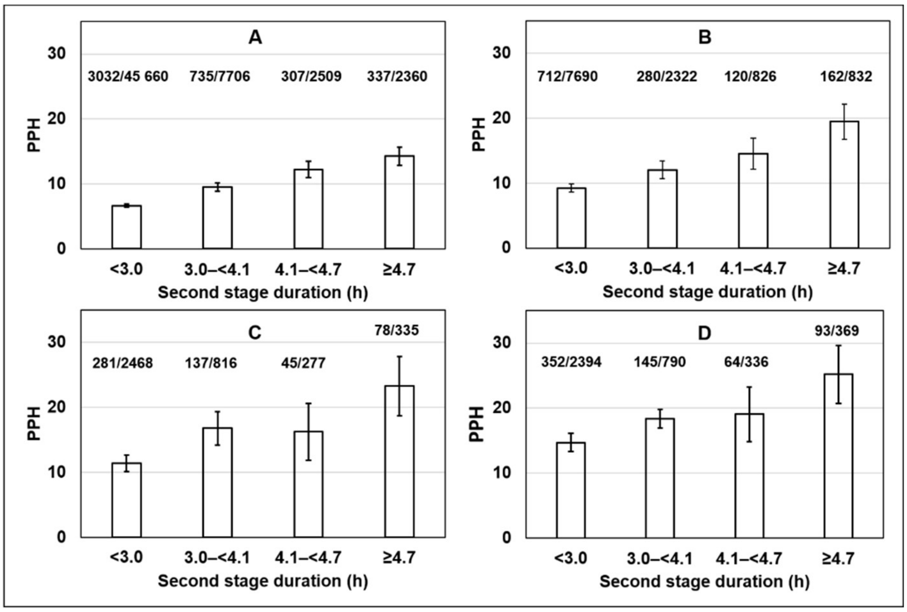

Supplementary Table S1. Unadjusted and adjusted risk ratios (RR) for PPH with increasing first stage labor duration in the study population with <50th percentile as reference, n=77,690

	First stage labor duration			
**Percentile of first stage labor duration**	<50th	50th – <70th	70th – <80th	≥80th
**First stage (h)**	<5.0	5.0 – <7.0	7.0 – <8.4	≥8.4
**Absolute risk n (%)**	2689 (6.9)	1337 (8.6)	764 (10.0)	2090 (13.4)
**Unadjusted RR (95% CI)**	1.00 (ref)	1.24 (1.17–1.33)	1.42 (1.31–1.53)	1.94 (1.84–2.05)
**Adjusted RR* (95% CI)**	1.00 (ref)	1.07 (1.00–1.14)	1.16 (1.07–1.25)	1.39 (1.31–1.48)

*Maternal age, maternal pre–pregnancy BMI, birth weight, oxytocin started before 10 cm cervical dilation, year of delivery and delivery clinic

n=3102/77,690 deliveries were excluded from the adjusted analysis due to missing data on confounder variables.

Supplementary Table S2. Unadjusted and adjusted risk ratios (RR) for PPH with increasing first stage labor duration in the study population with <60th percentile as reference, n=77,690

	First stage labor duration			
**Percentile of first stage labor duration**	<60th	60th – <80th	80th – <90th	≥90th
**First stage (h)**	<6.0	6.0 – <8.4	8.4 – <10.3	≥10.3
**Absolute risk n (%)**	3375 (7.2)	1415 (9.1)	895 (11.5)	1195 (15.4)
**Unadjusted RR (95% CI)**	1.00 (ref)	1.25 (1.18–1.33)	1.59 (1.49–1.71)	2.11 (1.99–2.25)
**Adjusted RR* (95% CI)**	1.00 (ref)	1.06 (1.00–1.13)	1.23 (1.14–1.32)	1.47 (1.37–1.58)

* Maternal age, maternal pre-pregnancy BMI, birth weight, oxytocin started before 10 cm cervical dilation, year of delivery and delivery clinic

n=3102/77,690 deliveries were excluded from the adjusted analysis due to missing data on confounder variables

Supplementary Table S3. Annual frequencies and rates (%) of postpartum hemorrhage (PPH) among singleton deliveries in the study population (n=77,690), nulliparous women at term with the infant in vertex position and spontaneous onset of labor between January 1, 2008 and June 15, 2020

	**n PPH/N (%)**
2008	230/3021 (7.9)
2009	512/6391 (8.0)
2010	552/6765 (8.2)
2011	571/6608 (8.6)
2012	603/6660 (9.0)
2013	631/6742 (9.4)
2014	596/6552 (9.1)
2015	570/6502 (8.8)
2016	613/6594 (9.3)
2017	549/6322 (8.7)
2018	573/6342 (9.0)
2019	575/6436 (8.9)
2020	296/2755 (10.7)

Supplementary Table S4. Mediation analysis of the association between a prolonged first stage of labor (≥75th percentile, ≥7.7 h) and postpartum hemorrhage (>1000 ml) in the study population (n=77,690) into natural direct effects and mediated effects, through prolonged second stage of labor (≥75th percentile, ≥3.0 h) or cesarean delivery

**Mediation analysis**	**Estimate (95% CI)**
Natural direct effect (odds ratio)	1.25 (1.15–1.36)
Mediated effect (odds ratio)	1.04 (1.02–1.07)
Proportion mediated (%)	18.5 (9.7–29.6)

CI, confidence intervals, constructed using the bootstrap method with 1000 samples.
Proportion mediated calculated as:
$100x[\text{Odds ratio}_{\text{Natural Direct Effect}} \text{ X } (\text{Odds ratio}_{\text{Mediated effect}} - 1)]/ [\text{Odds ratio}_{\text{Natural Direct Effect}} \text{ X Odds ratio}_{\text{Mediated effect}} - 1]$

Supplementary Table S5. Absolute risks of postpartum hemorrhage (PPH) and unadjusted and adjusted risk ratios (RR) for PPH with increasing first stage labor duration in the population of women with a recorded timepoint for 5 cm cervical dilation, n=39,717

	**First stage labor duration**			
**Percentile of first stage labor duration**	$<75^{th}$	$75^{th} – <90^{th}$	$90^{th} – <95^{th}$	$\geq 95^{th}$
**First stage (h)**	<8.0	8.0– <10.4	10.4 – <12.0	≥12.0
Absolute risk n (%)	2286 (8.0)	752 (10.8)	301 (13.6)	372 (19.8)
Unadjusted RR (95% CI)	1.00 (ref)	1.36 (1.26–1.47)	1.71 (1.53–1.91)	2.49 (2.25–2.74)
Adjusted RR* (95% CI)	1.00 (ref)	1.12 (1.03–1.21)	1.28 (1.14–1.45)	1.69 (1.52–1.89)

*Maternal age, maternal pre-pregnancy BMI, birth weight, oxytocin started before 10 cm cervical dilation, year of delivery and delivery clinic

n=1550/39,717 deliveries were excluded from the adjusted analysis due to missing data on confounder variables.

Supplementary Table S6. Characteristics of deliveries in the study population (n=77,690) compared to deliveries excluded (n=11,391) since their first recorded timepoint for cervical dilation was after 7 cm dilation.

**Characteristics**	**Study population n=77,690**	**First recorded timepoint >7cm n=11,391**
Maternal age median (IQR)	29 (26–32)	30 (27–33)
BMI median (IQR)	22.6 (20.8–25.0)	22.1 (20.6–24.4)
2nd stage duration median (IQR)	102 (54–179)	77 (41–143)
2nd stage ≥ 3 h	19277 (24.8)	1948 (17.1)
Post-term gestation (>42 weeks)	3311 (4.3)	229 (2.0)
Epidural analgesia	53,151 (68.4)	1611 (14.1)
Augmentation of labor with oxytocin started:		
No oxytocin	24,207 (31.2)	7427 (65.2)
< 10 cm	34,083 (43.9)	535 (4.7)
> 10 cm	19,400 (25.0)	3429 (30.1)
Mode of delivery		
SVD	63,712 (82.0)	10,477 (90.2)
OVD	11,615 (15.0)	991 (8.7)
CD (second stage)	2363 (3.0)	121 (1.1)
Birth weight≥4000 g	9414 (12.1)	974 (8.6)
PPH	6880 (8.9)	914 (8.0)

The study population includes women with a recorded timepoint for 5 cm cervical dilation or with a timepoint for 5 cm imputed using recorded timepoints at 3–4, 6–7 and 10 cm dilation. Results are presented as median (interquartile range) and n (column percent). IQR, interquartile range; BMI, body mass index; SVD, spontaneous vaginal delivery; OVD, operative vaginal delivery; CD, cesarean delivery.
